# Mg-based materials diminish tumor spreading and cancer metastases

**DOI:** 10.1016/j.bioactmat.2022.05.002

**Published:** 2022-05-10

**Authors:** Philipp Globig, Roshani Madurawala, Regine Willumeit-Römer, Fernanda Martini, Elisa Mazzoni, Bérengère J.C. Luthringer-Feyerabend

**Affiliations:** a Institute of Metallic Biomaterials, Helmholtz-Zentrum Hereon GmbH, 21502, Geesthacht, Germany; b Department of Medical Sciences, University of Ferrara, 44121, Ferrara, Italy

**Keywords:** Cancer, Osteosarcoma, Magnesium degradation, Cell migration, Cell invasion, Angiogenesis

## Abstract

Cancer metastases are the most common causes of cancer-related deaths. The formation of secondary tumors at different sites in the human body can impair multiple organ function and dramatically decrease the survival of the patients. In this stage, it is difficulty to treat tumor growth and spreading due to arising therapy resistances. Therefore, it is important to prevent cancer metastases and to increase subsequent cancer therapy success. Cancer metastases are conventionally treated with radiation or chemotherapy. However, these treatments elicit lots of side effects, wherefore novel local treatment approaches are currently discussed. Recent studies already showed anticancer activity of specially designed degradable magnesium (Mg) alloys by reducing the cancer cell proliferation. In this work, we investigated the impact of these Mg-based materials on different steps of the metastatic cascade including cancer cell migration, invasion, and cancer-induced angiogenesis. Both, Mg and Mg–6Ag reduced cell migration and invasion of osteosarcoma cells in coculture with fibroblasts. Furthermore, the Mg-based materials used in this study diminished the cancer-induced angiogenesis. Endothelial cells incubated with conditioned media obtained from these Mg and Mg–6Ag showed a reduced cell layer permeability, a reduced proliferation and inhibited cell migration. The tube formation as a last step of angiogenesis was stimulated with the presence of Mg under normoxia and diminished under hypoxia.

## Highlights

• Magnesium (Mg)-based material degradation decrease cell migration and invasion of an osteosarcoma coculture.
• Mg-based material degradation products reduce cancer-induced angiogenesis at an early stage.
• These materials may reduce secondary tumor formation and metastases.

## Graphical abstract

**Figure:**
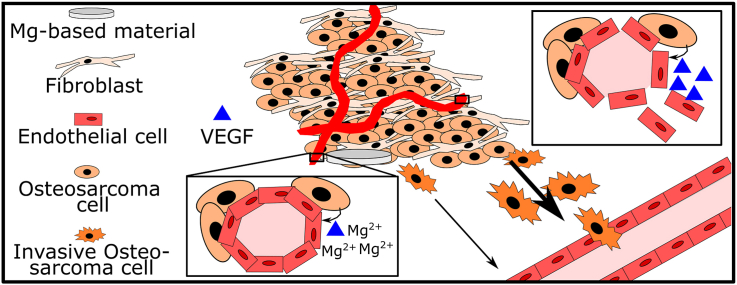

## 1 Introduction

“Cancer metastases” describes the detachment of single invasive cancer cells from the primary tumor that migrate through the circulation and form new, secondary tumors at distant sites of the body. The diagnosis of metastases dramatically decreases the cure and survival rate of patients and accounts for more than 90% of the cancer-related deaths [1]. Therefore, it is crucial to prevent cancer dissemination by inhibiting the development of metastases or direct treatment against secondary tumors. Cancer metastases are conventionally treated by radiation and chemotherapy to eliminate residual disseminated cancer cells from the primary tumor sites. These treatment approaches are only totally successful for a minority of patients and provoke severe side reactions [2]. Therefore, novel local treatment strategies should be used in future to increase therapy success and the patient's everyday life, by preventing tumor dissemination [3]. Such a novel approach may be the treatment with specifically for this purpose designed degradable magnesium (Mg) materials. Mg-based materials in general are already in clinical use as screws and pins in orthopedics or as degradable stents in cardiovascular applications [[4], [5], [6], [7]]. Aside from this, Mg-based materials have been recently linked to an increased cytotoxicity towards cancer cells [[8], [9], [10]]. Due to their biocompatibility and degradability, Mg-based materials can be exploited as a drug release system with a respective coating of an active agent. Furthermore, the degradation of Mg-based materials provokes surface-near effects such as the increase in pH, in osmolality, and an elevated hydrogen gas evolution, which are tunable via the degradation rate [11]. Those surface-near effects additionally hold the potential to directly target cancer cells and reduce the tumor growth, as previously published [12]. The introduction of silver (Ag) or other alloying elements can influence the material's degradation rate and biological response. Such biological responses may include an increased cytotoxicity or in case of Ag antibacterial activity [13,14]. However, Mg^2+^ ion repletion after Mg^2+^ deficiency was also recently reported to increase the tumor growth in mice [15], while a Mg^2+^ deficiency was associated with a proinflammatory response [16], activation of vascular endothelial cells [17] and metastases [15]. This hints to a dual function of Mg^2+^ in tumor development, with a promoting function at early stages and inhibiting effects at late-stage solid cancers. Whether this is also valid for Mg-based materials is unknown. Here, we examined the influence of Mg-based materials on the cancer-induced angiogenesis, as an early step of tumor progression, and invasion and metastases, representative for later stages of the so-called “metastatic cascade”. This publication should also introduce methods that can be used to investigate the influence of different Mg-based materials (size and geometry-independent) on the cancer cell migration and invasion and the cancer-induced angiogenesis. To meet those requirements, we decided to use an indirect experimental set-up. In turn, this hampers the impact of the surface-near effects, which cannot taken into account with this set-up.

The metastatic cascade is a model by Fidler to outline the complex phenomenon of disseminated cancer cells [18]. This model describes that cancer metastasizes in a multistep process, whereas every step has to be successfully passed to reach to the following step. Thus, the probability of metastasizing tumors is very low and was valued at <0.1% by animal melanoma studies [19]. In clinics, a proportion of 15–20% of osteosarcomas show metastases at diagnosis [20]. However, targeting cancer metastases by alternative treatment strategies is relevant due to the high mortality and morbidity that are emanating from aggressive metastases. Cancer cells that initiate the metastatic cascade first have to detach from their adjacent neighbor cells. During this detachment the cancer cells change from an epithelial to a mesenchymal phenotype, called epithelial-mesenchymal transition (EMT), by changing the gene expression profile. While E-cadherin is downregulated, vimentin and matrix metalloproteinases (MMP) are upregulated to increase motility and invasiveness [21]. The interaction of the cancer cells with host cells in the tumor microenvironment (TME) is also essential for the ongoing metastatic cascade for examples the communication with fibroblasts and macrophages. Those cells are exploited by the cancer cells to further release MMP-2 and MMP-9 to degrade the surrounding extracellular matrix (ECM) that cancer cells can reach vasculature [22]. There, cancer cells intravasate into the blood stream, survive in circulation and extravasate to the secondary site, where the cancer cells are then challenged to survive and proliferate in the new environment [23].

**Fig. A1:**
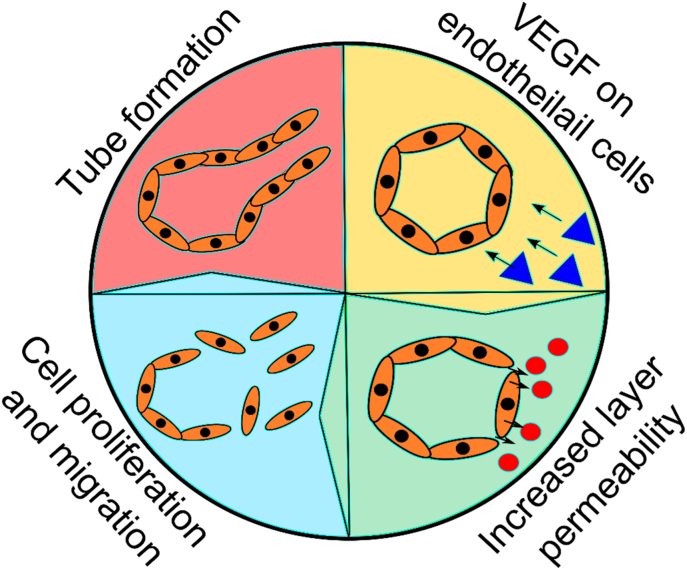
Cancer-induced angiogenesis. Endothelial cells are influenced by VEGF (blue triangle), which results in a leaky cell layer. This ameliorates the release of plasma proteins (red circles).

Due to the cancer cell reliance on the vasculature as a transport system, cancer metastases are linked to another important prerequisite of tumor progression: cancer-induced angiogenesis [24]. Angiogenesis is the formation of new blood vessels from preexisting ones and is essential to supply the cancer with nutrients and oxygen. Thus, the process to extend the vasculature is triggered within a growing tumor where regions in the tumor core become hypoxic (<3% O_2_) and poor in nutrients [25]. The vascular endothelial growth factor (VEGF) is one of the most important mediators of angiogenesis and is involved in almost all steps [26]. First, the endothelial cell layer that lines the blood vessels becomes leaky, which allows the extravasation of e.g. MMPs. The permeable blood vessel, in turn, is also advantageous for intravasation and extravasation of invasive cancer cells, further linking angiogenesis with cancer metastases. Subsequently, endothelial cells proliferate and migrate to extend existing vessels and reconnect to adjacent endothelial cells to form new extended lumens [27] (see figure A1)

In this study, we investigated the influence of Mg-based materials on different steps of the metastatic cascade. A previously described osteosarcoma-fibroblast coculture was used to show the impact of Mg materials on cell migration and invasion. Moreover, human umbilical vein endothelial cells (HUVEC) were exposed to Mg material degradation products containing media to examine the effects on cancer-induced angiogenesis.

## 2 Materials and methods

### 2.1 Cell culture

Saos-eGFP were obtained by genetic engineering of Saos-2 (human osteosarcoma) and kindly provided by Prof. Mauro Tognon (Department of Medical Sciences, University of Ferrara, 44121 Ferrara, Italy). A detailed description of the cell line engineering can be found in the publication by Morelli et al. [28]. Red fluorescent (RF) fibroblasts were purchased from Innoprot (Innoprot, Derio, Spain) as modified primary human dermal fibroblasts expressing the fluorescent protein FP602. Saos-eGFP as well as RF Fibroblasts were grown in Dulbecco's Modified Eagle Medium GlutaMAX-I (DMEM GlutaMAX-I; Life Technologies, Darmstadt, Germany) with a 10% fetal bovine serum (FBS, Merck KGaA, Darmstadt, Germany) supplementation under cell culture conditions (37 °C, 5% CO_2_, 95% relative humidity (rH)).

Human umbilical cords were kindly provided by the Agaplesion Bethesda Krankenhaus Bergedorf (Hamburg, Germany) and human umbilical vein endothelial cells were isolated and characterized as described by Xu et al. with the ethical approval of the Ethik Kommission der Ärztekammer Hamburg (PV4058) [29]. The cells were maintained in endothelial cell growth medium 2 (ECGM - Promocell, Heidelberg, Germany) and used up to passage 6.

### 2.2 Preparation of conditioned media

The cancer-induced angiogenesis was investigated with HUVEC that were incubated with conditioned media obtained from seeding Saos-eGFP and RF Fibroblasts as coculture or monocultures on Mg-based materials. First, this avoided the combination of opaque materials and a third cell line, and second, the relevance of the results was ascertained.

Pure Mg (99.95%) and Mg–6Ag (6 wt% Ag) disks were produced at Helmholtz-Zentrum hereon (Helmholtz-Zentrum hereon GmbH, Geesthacht, Germany) as described in a previous publication [12] (see Table 1 for chemical composition). The materials were surface treated, cleaned and sterilized prior to cell seeding. For surface treatment, Mg and Mg–6Ag materials were wet-ground (Saphir 360 from ATM GmbH, Mammelzen, Germany) using SiC 2500 grid paper (Starcke GmbH & Co.KG, Melle, Germany). Then the materials were serial immersed in n-hexane, acetone and ethanol (all Merck KGaA, Darmstadt, Germany) in a beaker and ultrasonically cleaned (Branson 1210, Branson Ultrasonics, Danbury, USA), each solution for 20 min. The disks were further sterilized in the ultrasonic bath using 70% ethanol for 20 min and preincubated in a 24-well plate (Greiner Bio-One International GmbH, Kremsmünster, Austria), in a volume of 2 mL of complete DMEM cell culture medium, for 24 h. Afterwards, Saos-eGFP and RF Fibroblasts were detached from cell culture flasks using 0.05% trypsin-EDTA (Life Technologies GmbH, Darmstadt, Germany) for 5 min at 37 °C. The cells were counted (CASY Cell Counter, Roche Diagnostics GmbH, Mannheim, Germany) and Saos-eGFP and RF Fibroblasts were mixed as a 1:1 coculture or used as monocultures with a seeding density of 250.000 cells/mL. Afterwards, 40 μL (10,000 cells) were carefully applied onto the surface of Mg, Mg–6Ag or glass slides. To allow the cells to adhere to the surfaces, the well-plates were incubated at 37 °C for 20 min. Then, 2 mL of complete DMEM cell culture medium were added to each sample and incubated under cell culture conditions with normoxia (20% O_2_) or hypoxia (3% O_2_).

**Table 1:** Chemical composition of the here used Mg-based materials (weight %). Ag: silver, Al: aluminum, Cu: copper, Fe: iron, Mg: magnesium, Ni: nickel.

	Mg	Ag	Al	Cu	Fe	Ni
Mg	99.94	<0.00005	0.013–0.016	0.0002–0.0003	0.0048–0.0049	<0.0002
Mg–6Ag	≈94	5.94–6.34	<0.0100	0.0013–0.0014	0.0019–0.0021	0.009–0.0010

**Fig. A2:**
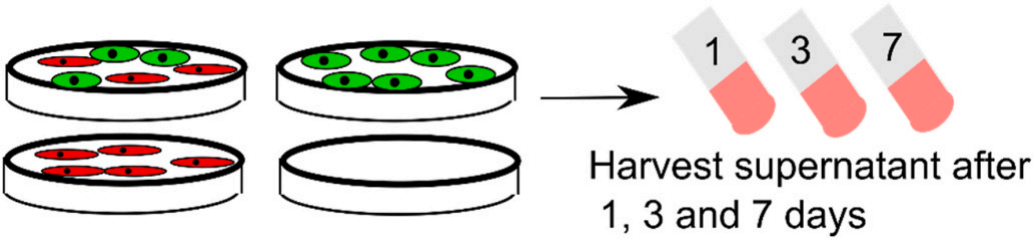
Scheme of conditioned media production. Saos-eGFP (green) and healthy fibroblasts (red) were seeded on the different materials (Mg, Mg–6Ag, glass slides) in different culture combinations (monocultures, coculture).

Figure A2 (supplementing material) shows the scheme of conditioned media harvesting one, three and seven days after cell seeding. Fresh medium was added after every harvest step.

The Mg concentration of the conditioned media was measured via atomic absorption spectroscopy (AAS). The conditioned media were diluted 1:250 using 1% (w/v) nitric acid (HNO_3_, Merck KGaA, Darmstadt, Germany) in ultrapure H_2_O. Afterwards, Mg concentration was determined with a flame AAS (Agilent 240 AA, Agilent Technologies, Waldbronn, Germany) at a wavelength of 285.2 nm (emission spectrum of Mg) and quantified using a calibration curve ranging from 0.05 to 1 mg/L Mg (AAS standard solution ROTI®star Carl Roth Karlsruhe, Germany) as described in our previous work [12].

The Mg-based materials’ degradation rate was measured by weight loss. Therefore, the weight of the samples was determined before and after the immersion. Afterwards, the samples were immersed in chromic acid (180 g/L in distilled water, VWR International, Darmstadt, Germany) to get rid of the residual degradation layer. More details can be found in a previous publication [12].

### 2.3 Determination of cell migration

To monitor cell migration, the proliferation of Saos-eGFP and RF Fibroblasts was inhibited by mitomycin. Therefore, the cells were separately starved overnight in serum-free DMEM medium before incubating Saos-eGFP with 10 μg/mL and RF Fibroblasts with 50 μg/mL mitomycin c (MMC, from Streptomyces, Sigma-Aldrich Chemie GmbH, Munich, Germany) for 2 h at 37 °C. Subsequently, the MMC solution was removed, and residuals washed away with three PBS washing steps.

The cell migration was investigated with a wound healing assay. Therefore, Mg-based materials were cleaned and sterilized as described in chapter 2.2. Concomitant, proliferation inhibited Saos-eGFP and RF Fibroblasts were seeded into 12-well plates (Greiner Bio-One International GmbH, Kremsmünster, Austria) as a 1:1 coculture or as monocultures with a total number of 50,000 cells per well. The cells were allowed to adhere to the well plates for 24 h. Subsequently, a 20–200 μL pipette tip was used to vertically scratch the confluent cell monolayer of every well. The cells were washed once with PBS to remove detached cells and microscopic images of three randomly chosen positions per well alongside the scratch were taken. An inverse fluorescence microscope (Eclipse Ti–S; Nikon GmbH, Düsseldorf, Germany) with a suitable filter set was used to visualize the cell fronts besides the scratch with Saos-eGFP (FITC) and RF Fibroblasts (TexasRed). Afterwards, the Mg-based materials were transferred into 12-well inserts (3 μm pores, high density, translucent, Corning Inc., Corning, NY, USA) and placed into the 12-well plates above the cells. The procedure of fluorescence image acquisition was repeated after 24 and 48 h with the exact same chosen positions. The scratch area of the different image sections was quantified using ImageJ (Rasband, W.S.,ImageJ, U.S. National Institutes of Health, Bethesda, MD, USA, “https://imagej.nih.gov/ij/", 1997–2018). The progression of the cell front convergence was analyzed by the size of relative cell-free areas 24 and 48 h (relative to the initial time point) after “wound” setting.

The experimental set-up for endothelial cell migration was slightly changed. HUVEC were seeded into 24-well plates and the proliferation was inhibited with 10 μg/mL MMC. After producing the scratch, the medium was changed to the conditioned media. Brightfield images were taken with the inverse microscope and analyzed as described in the previous paragraph.

### 2.4 Determination of cell invasion

**Fig. 1:**
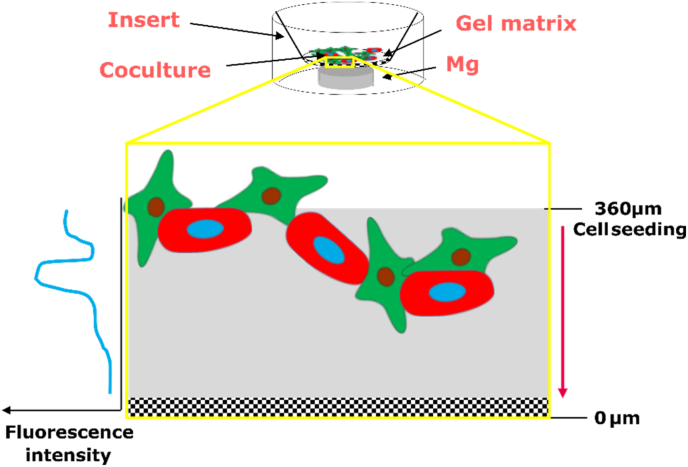
Scheme of the experimental set-up to investigate the cell invasion.

Cell invasion was investigated with a modified Boyden chamber experimental set-up (Fig. 1).

PureCol™ EZ Gel solution (Sigma-Aldrich Chemie GmbH, Munich, Germany) was thawed on ice and diluted 1:4 in serum-free DMEM. From this solution, 50 μL were applied to each 24-well inserts (8 μm pores, Sarstedt AG & Co. KG, Nümbrecht, Germany), which were then placed into a 24-well plate with a carrier plate (Thermo Fisher Scientific, Waltham, MA, USA). The gel was allowed to solidify at 37 °C for 90 min followed by the cell seeding. Saos-eGFP and RF Fibroblasts were prepared as a 1:1 coculture or as monocultures in DMEM supplemented with 0.5% FBS. A volume of 250 μL of these cell suspensions that contained a total number of 50,000 cells was then added onto the gel surface. Prior to this, Mg and Mg–6Ag were ground, cleaned, and sterilized as described in chapter 2.2. Those materials were transferred to the bottom of the 24-well plates (lower chamber) in complete DMEM cell culture medium, while the gel/cell-containing inserts were placed on top. After incubation for 72 h, cells were visualized at the confocal laser scanning microscope (cLSM, DM6000 CS, Leica, Wetzlar, Germany) by turning the inserts upside down on a microscope slide. Fluorescence intensities of Saos-eGFP and RF Fibroblasts were detected in z-stacks with 10 μm steps. Additionally, invaded cells were visualized with a 0.05% crystal violet solution (Merck KGaA, Darmstadt, Germany) and an inverse microscope (Eclipse Ti–S; Nikon GmbH, Dusseldorf, Germany) and counted on the underside of the insert membrane. The cell invasion was shown by the fluorescence intensities dependent on the distance to the membrane, and the number of crystal violet stained cells (five randomly chosen positions on the membrane).

### 2.5 Determination of molecular markers for invasion and angiogenesis

MMP-2, MMP-9 and TIMP-1 release was measured in the supernatant after conditioned media production (chapter 2.2) by enzyme-linked immunosorbent assay (ELISA). VEGF concentration was quantified in the supernatant of HUVEC incubated with conditioned media. All cytokines were detected with a DuoSet ELISA (R&D Systems, Minneapolis, MN, USA) as described by the manufacturer. Washing steps (0.05 %Tween 20 in PBS) were performed with the respective voluminal according to the manufacturer's suggestion with a wash station (Bioplex Pro Wash station, Biorad, Hercules, CA, USA). In brief, the capture antibody was diluted in PBS and immobilized in 96-well plates (High binding, Sarstedt AG & Co., Nürnbrecht, Germany). Then, plates were blocked with reagent diluent (1% bovine serum albumin, BSA in PBS) and 100 μL supernatant or standard were added for 2 h at room temperature. After adding the detection antibody and the horse radish peroxidase conjugated streptavidin, the substrate solution initiated the color reaction (blue). This color reaction was stopped with H_2_SO_4_ (yellow) and the color change was detected with a plate reader (Sunrise™ Tecan microplate reader; Tecan, Männedorf, Switzerland) set to 450 nm. MMP-2, MMP-9 and TIMP-1 concentrations were normalized to the cell numbers of cancer cells and fibroblasts. The VEGF concentration in the HUVEC supernatant was put in relation to medium control.

### 2.6 Assessment of HUVEC layer permeability

The HUVEC layer permeability was assessed by the amount of FITC-dextran (Sigma-Aldrich Chemie GmbH, Munich, Germany) that passed though the HUVEC layer. A total number of 50,000 HUVEC were seeded in 24-well transwell inserts (0.4 μm pores, Thermo Fisher Scientific, Waltham, MA, USA). The inserts were placed into a respective well-plate with carrier plate (Thermo Fisher Scientific, Waltham, MA, USA), splitting the set-up into an upper and a lower compartment. The HUVEC were incubated with ECGM for 72 h to form a confluent monolayer. Afterwards, the medium was changed to the conditioned media. The permeability was then measured three and seven days after the medium change. Therefore, 25 μg/mL FITC-dextran were applied to the upper compartment and the plate was shaken (MaxQ™ 4000 Benchtop Orbital Shakers, Thermo Fisher Scientific, Waltham, MA, USA) at 60 rpm for 60 min with an aluminum foil to protect the plate from light. Subsequently, the lower compartment was transferred to 96-well plates (Greiner Bio-One International GmbH, Kremsmünster, Austria) and fluorescence was measured in triplicates (100 μL) with a Victor^3^ multilabel plate reader (PerkinElmer, Inc., Waltham, MA, USA) at an excitation wavelength of 485 nm and emission wavelength of 535 nm. Furthermore, samples with a known FITC-dextran concentration were also measured to quantify the unknown samples with a standard curve.

### 2.7 HUVEC proliferation

HUVEC proliferation was monitored by cell nuclei staining. The HUVEC were seeded in a density of 2000 cells/well and were allowed to adhere in 96-well plates (Greiner Bio-One International GmbH, Kremsmünster, Austria). Subsequently, the HUVEC were stained with 2 μg/mL Hoechst33342 (Thermo Fisher Scientific, Waltham, MA, USA) diluted in serum-free medium at 37 °C for 15 min. The initial cell nuclei fluorescence was measured with a Victor^3^ multilabel plate reader (PerkinElmer, Inc., Waltham, MA, USA) at an excitation wavelength of 355 nm and emission wavelength of 460 nm. The proliferation monitoring was then started with the change of medium to the different conditioned media. The fluorescence measurement was repeated three and seven days after the medium change and resulting fluorescence intensities related to these of the initial measurement.

### 2.8 Tube formation assay

Endothelial cell tube formation was determined on a Geltrex® LDEV-free reduced growth factor basement membrane matrix (Thermo Fisher Scientific, Waltham, MA, USA). Therefore, the matrix was thawed overnight at 4 °C. A volume of 15 μL of this matrix were applied into each well of 96-well plates. The plates were incubated under cell culture conditions for 30 min to allow the matrix to solidify. HUVEC (10,000 cells) were seeded on the matrix, allowed to adhere for 30 min and medium was changed to conditioned medium. After 6 h, the conditioned media were replaced by a calcein staining solution (1 μM calcein-AM, Thermo Fisher Scientific, Waltham, MA, USA) and incubated for 30 min under cell culture conditions. Subsequently, microscopic images were taken (Eclipse Ti–S; Nikon GmbH, Dusseldorf, Germany) and the resulting images were analyzed with the ImageJ plugin “Angiogenesis analyzer” (Rasband, W.S.,ImageJ, U.S. National Institutes of Health, Bethesda, MD, USA, “https://imagej.nih.gov/ij/", 1997–2018).

### 2.9 Statistical analysis

Data regarding the invasion and metastases was obtained from two independent experiments with two samples in triplicates (n = 12) if not stated otherwise. The results from the cancer induced angiogenesis were obtained from two independent experiments with three samples or in triplicates (n = 6) if not stated otherwise. All data is shown as the arithmetic mean ± standard deviation (SD). The statistical analysis was performed using Prism 6 (GraphPad Software, La Jolla, CA, USA): Comparison of cell migration (Saos-eGFP, RF Fibroblasts, HUVEC), MMP and TIMP-1 concentration and permeability, proliferation, migration, and tube formation of HUVEC with a two-way ANOVA with Tukey's multiple comparison test. The cell invasion with the z-stacked images was analyzed using an ordinary one-way ANOVA with Dunnett's multiple comparison test.

## 3 Results

### 3.1 Influence of Mg-based materials on cancer metastases

#### 3.1.1 Mg-based materials reduced cell migration and invasion

To measure the cell migration and invasion, the cells were seeded with the materials, and therefore, the cells were exposed to the material's degradation kinetics and surface-near effects.

**Fig. 2:**
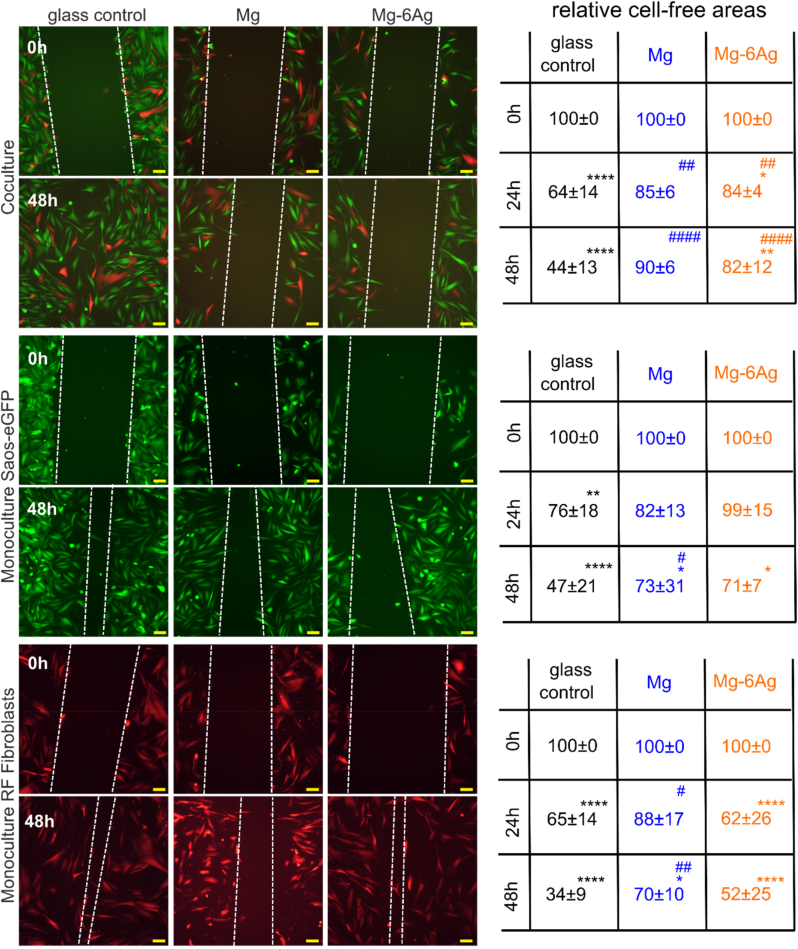
Cell migration influenced by Mg and Mg–6Ag under normoxia. Microscopic images of Saos-eGFP (green) and RF Fibroblasts (red) in coculture or monocultures with the initial wound (0 h) and after 48 h. The white dotted lines symbolize the cell fronts. Scale bar is 100 μm. The cell-free areas after 24 h and 48 h were quantified in relation to the initial cell-free area. Relative cell-free areas are shown as the mean ± SD from two experiments with two samples and three randomly chosen positions (n = 12). Statistics: two-way ANOVA (Mg, Mg–6Ag compared to glass control = #; 24 h, 48 h compared to 0 h = *) with Tukey's multiple comparison test. One symbol = p < 0.05; two symbols = p < 0.01; three symbols p < 0.001; four symbols = p < 0.0001.

**Fig. A3:**
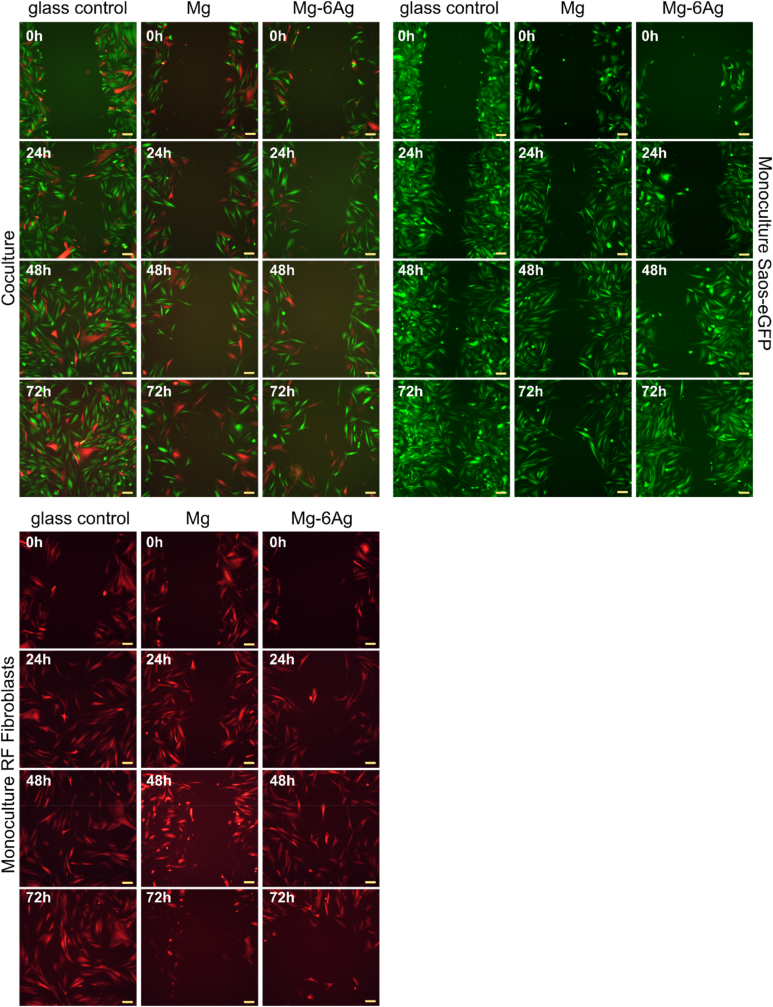
Migration assay with Mg-based materials. This figure is equivalent to Fig. 2 with time points between 0 h and 72 h. Scale bar is 100 μm.

Fig. 2 shows the influence of slow degrading Mg and Mg–6Ag (compare Table 2 for mean degradation rates and Mg and Ag content in the supernatant) on tumor and healthy cell migration in monocultures and coculture under normoxia up to 48 h. The microscopic images of the time points between 0 h and 72 h are shown in Figure A3 in the appendix.

**Table 2:** Mean degradation rates and magnesium and silver content in the supernatant during immersion of the here used Mg-based materials. Values are shown as the mean ± SD. MDR: mean degradation rate. Table is adapted from Ref. [30].

Immersion time (d)	Mg			Mg–6Ag		
	MDR (mm/a)	c_Mg_ (mM)	c_Mg-6Ag_ (μM)	MDR (mm/a)	c_Mg_ (mM)	c_Mg-6Ag_ (μM)
1	0.73 ± 0.25	2.46 ± 0.52	–	0.63 ± 0.31	1.25 ± 0.36	0.25 ± 0.20
3	0.25 ± 0.02	3.75 ± 0.83	–	0.29 ± 0.06	2.13 ± 0.31	0.17 ± 0.08
7	0.20 ± 0.06	4.02 ± 0.48	–	0.18 ± 0.07	2.54 ± 0.74	0.24 ± 0.10

The coculture and monoculture samples showed a significant wound closure, when they were incubated with the non-degrading glass control. Compared to the glass control, the migration inhibition was significantly (Mg) or less significant (Mg–6Ag) with the Mg-based materials in the coculture within 48 h. The coculture that was incubated with Mg and Mg–6Ag showed a significant reduction of cell migration compared to the glass control already after 24 h. For the monoculture of Saos-eGFP, that were incubated as a monoculture with Mg-based materials only showed a significant wound closure after 48 h. Furthermore, only the cancer cells incubated with pure Mg showed a significantly larger cell-free area after 48 h compared to the glass control. The cell migration provoked by Mg–6Ag in the healthy cells did not significantly differ compared to the glass control. However, the cell migration of healthy cells that were incubated with slow degrading pure Mg was significantly reduced compared to the glass control already after 24 h.

**Fig. 3:**
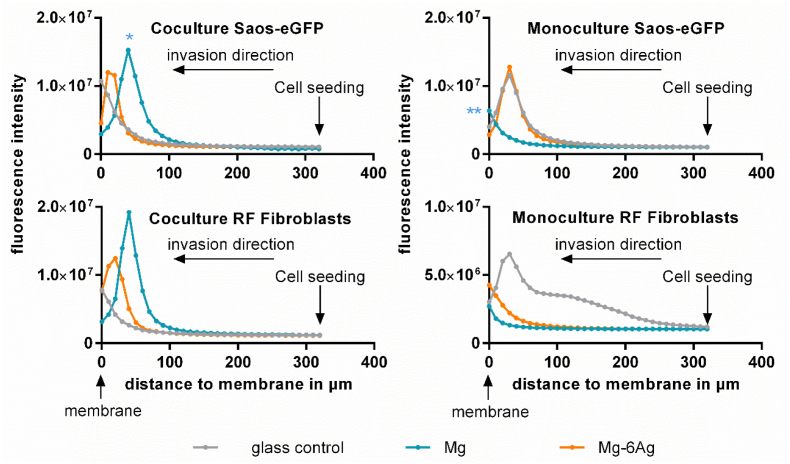
Cell invasion influenced by Mg and Mg–6Ag. Cells invaded through an ECM mimetic gel layer from the point of cell seeding (320 μm) to the membrane (0 μm). Fluorescence intensities of representative z-stack (10 μm steps) images are shown. Statistics: ordinary one-way ANOVA with Dunnett's multiple comparison test (n = 12); * = p < 0.01, ** = p < 0.01.

As part of the metastatic process, the influence of Mg and Mg–6Ag materials on the invasive potential of the coculture was investigated. Fig. 3 shows the change of the maximal fluorescence intensity representing the movement of the fluorescent cancer and healthy cells through an ECM mimetic gel layer from the level of cell seeding (320 μm) to the membrane (0 μm). Saos-eGFP that were incubated in coculture with pure Mg showed a significant reduction of cell invasion compared to cells with the glass, as it can be seen by the maximum fluorescence intensity that is further away from the membrane. This was entirely different in the monocultures. Here, the invasion of cancer and healthy cells that were incubated with pure Mg seemed to be increased compared to the control on glass.

**Fig. 4:**
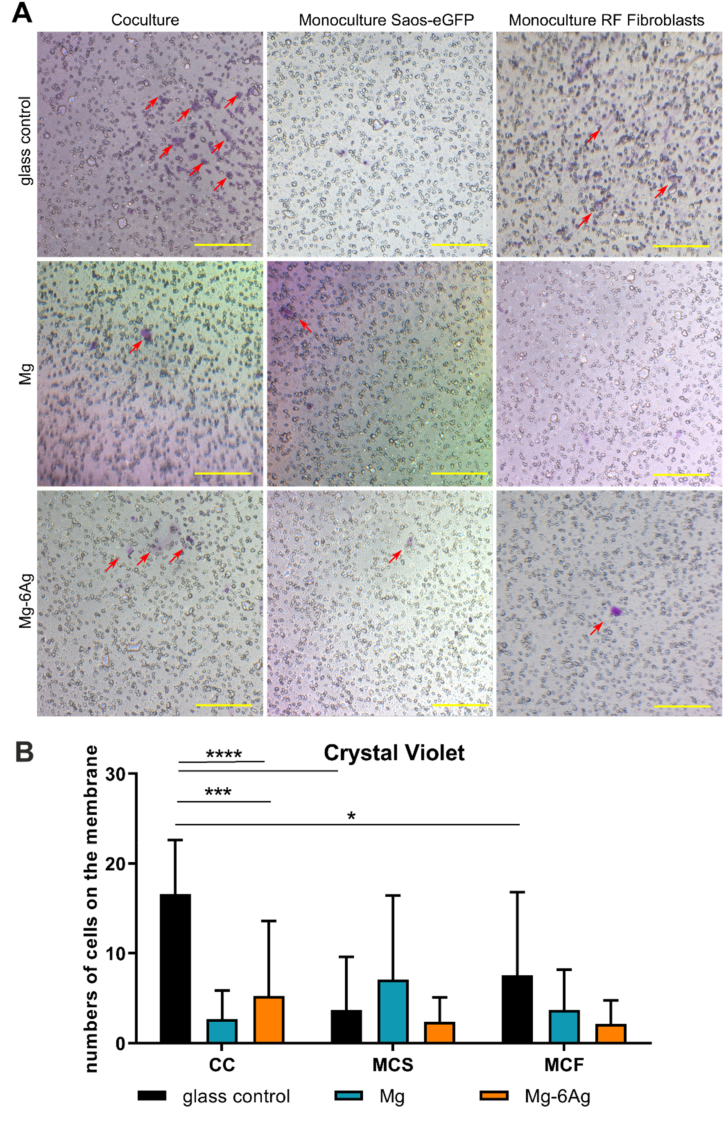
Visualization of invasive cells. (A) Cells that invaded the ECM mimetic gel layer and crossed the membrane were stained with crystal violet. Red arrows on representative images indicate invaded cells, which were quantified (red numbers). Scale bar is 100 μm. (B) Quantitative analysis of images. Statistics: two-way ANOVA (Mg, Mg–6Ag compared to glass control = *) with Tukey's multiple comparison test (n = 12). * = p < 0.05; *** = p < 0.001; **** = p < 0.0001.

Fig. 4 quantifies the cells that invaded through the ECM-like gel and through the pores to the other side of the membrane. Overall, it confirmed the results observed in Fig. 3. The number of invasive coculture cells was significantly reduced with both, Mg and Mg–6Ag, compared to the glass control. However, the number on invaded Saos-eGFP and RF Fibroblasts in monocultures were also significantly reduced, when incubated with the glass control compared to the respective coculture.

To summarize this, the migration and invasion of tumor cells and healthy fibroblasts in coculture, which were incubated with slow degrading Mg-based materials, were significantly reduced. However, the results obtained from monocultures differed remarkably from the ones of the coculture.

#### 3.1.2 Mg-based materials increased MMP-2, MMP-9 and TIMP-1 release

**Fig. 5:**
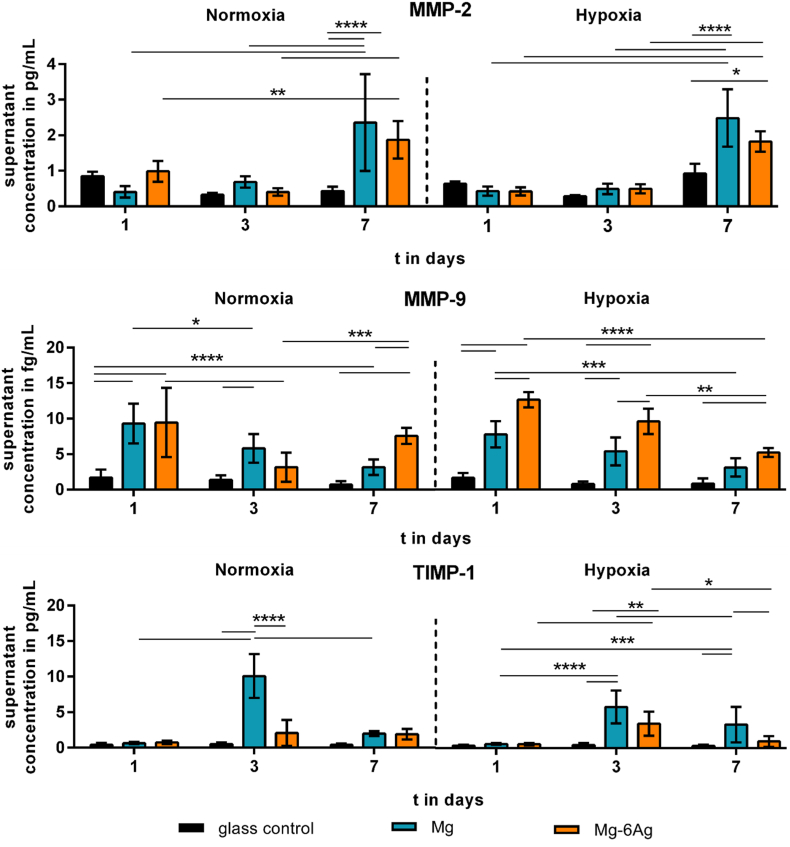
The impact of Mg-based materials on metastases-associated cytokine release. MMP-2, MMP-9 and TIMP-1 was quantified in the supernatant of migrating cells of the coculture and normalized to the cell numbers. Normalized cytokine concentrations are shown as the mean ± SD from two experiments with two samples in duplicates (n = 12). Statistics: two-way ANOVA (materials, time points) with Tukey's multiple comparison test. * = p < 0.05; ** = p < 0.01; *** = p < 0.001; **** = p < 0.0001.

During the metastatic cascade, cancer cells detach from their primary site and move through the tissue to the endothelial cells and circulation. The release of matrix metalloproteinases leads to the degradation of ECM which is a prerequisite for the release and mobility of single cancer cells. Fig. 5 shows the quantification of MMP-2, MMP-9 and their inhibitor TIMP-1 that were released into the supernatant by the migrating coculture normalized to the cell numbers on the different materials. The normalized MMP-2 release from the coculture on Mg and Mg–6Ag significantly increased up to day 7 and was significantly higher compared to the glass control on that day. This was observed irrespective of the oxygen level.

The normalized MMP-9 expression in the coculture was also significantly higher under the influence of Mg-based materials compared to the non-degrading glass control. While the normalized MMP-9 expression remained constant on glass, the normalized concentration declined from day 1 to day 7 under normoxia and hypoxia.

The normalized TIMP-1 concentration in the supernatant increased to a maximum at day 3 and fell afterwards for cells seeded on Mg. This effect was more pronounced under normoxia than hypoxia. The normalized TIMP-1 concentration at day 3 was significantly higher for pure Mg compared to Mg–6Ag and the glass control, under both conditions.

### 3.2 The impact of Mg degradation on cancer-induced angiogenesis

#### 3.2.1 Mg-based materials reduced angiogenesis initiation

**Fig. 6:**
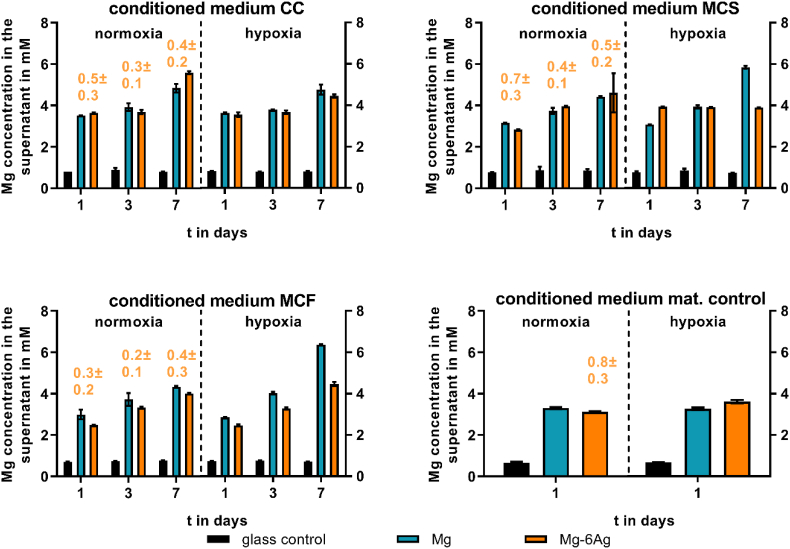
Supernatant Mg and Ag concentration of conditioned media. Saos-eGFP and RF Fibroblasts were seeded as a 1:1 coculture (CC) or monocultures (Saos-eGFP: MCS, RF Fibroblasts: MCF) on Mg, Mg–6Ag or glass (glass control). Furthermore, material without cells served as a mat. control. After one, three and seven days, conditioned medium was harvested, and Mg (in mM) and Ag (orange numbers, in μM) concentration were measured as described previously [12].

Cancer-induced angiogenesis was investigated by an indirect model using HUVEC with conditioned media prepared from cocultures of osteosarcoma cells and fibroblasts grown on Mg and Mg–6Ag material surfaces (1, 3 and 7 days). This experimental set-up reduced the difficulties to handle and monitor three cell lines on opaque Mg-based materials, while ensuring a certain relevance of the results. The magnesium and silver concentrations of the conditioned media are shown in Fig. 6.

Conditioned media that were free from Mg-based materials showed a supernatant Mg concentration around 0.8 mM, which is the preset concentration in the cell culture medium. In contrast to this, conditioned medium that was prepared with Mg-based materials increased the supernatant Mg concentration to 3–6 mM irrespective of the cell type. This concentration elevated with the increased degradation time and seemed to be not O_2_ dependent. In contrast to this, Ag concentrations did not seem to increase with the degradation time.

**Fig. 7:**
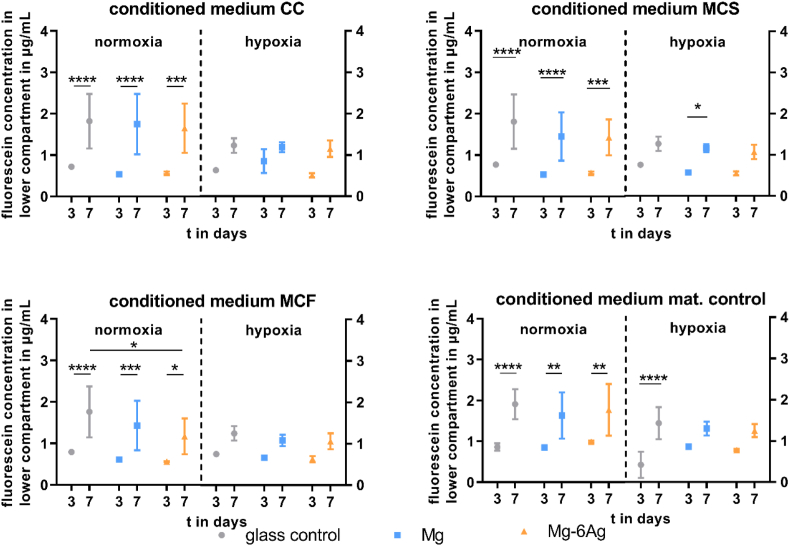
Endothelial cell layer permeability with different conditioned media. HUVEC were incubated with conditioned media from the coculture (CC) or Saos-eGFP (MCS) and RF Fibroblasts (MCF) in monocultures on Mg-based materials or material without cells. Fluorescein-dextran concentrations (in μg/mL; quantified with a standard curve) are shown as the mean ± SD from two experiments with one triplicate (n = 6). Statistics: two-way ANOVA (materials, time points) with Tukey's multiple comparison test. * = p < 0.05; ** = p < 0.01; *** = p < 0.001; **** = p < 0.0001.

Cancer-associated angiogenesis is initiated by degradation of the surrounding ECM and loosening of binding to adjacent endothelial cells. Hereby, the permeability of the endothelial cell layer increases to facilitate the extravasation of proteins. Fig. 7 describes the permeability of the HUVEC layer dependent on conditioned media with or without Mg-based materials. The permeability of interendothelial junctions significantly increased from day 3 to day 7 under normoxia. However, this permeability increase was not as pronounced under hypoxia. Additionally, the permeability tended to be slightly higher, when HUVEC were incubated with cell-seeded conditioned medium from the glass control (CC, MCS, MCF) compared to the respective conditioned media from Mg-based materials at day 7.

VEGF is one of the most important mediators of angiogenesis and is responsible for the formation of new blood vessels through stimulation of endothelial cell proliferation and migration. Therefore, the VEGF concentration in the supernatant of HUVEC that were incubated with the conditioned media was measured (Table 3, Appendix Table A4). The incubation of Mg-based materials and the glass control with the coculture or the fibroblasts in monoculture did not provoke a significantly different VEGF release. In contrast to this, the incubation of HUVEC with the conditioned medium of the monoculture of cancer cells on glass under hypoxic conditions led to a significant increase of the normalized VEGF concentration at day 3 (Table 3).

**Table 3:** VEGF release under hypoxia. HUVEC were incubated with conditioned media from the coculture (CC) or Saos-eGFP (MCS) and RF Fibroblasts (MCF) in monocultures on Mg-based materials or material without cells. Fold changes of VEGF concentration (relative to cell culture medium) are shown as the mean ± SD from two experiments with three samples (n = 6). Statistics: one-way ANOVA (sample vs medium control) with Dunn's multiple comparison test. **** = p < 0.0001.

	Day	conditioned medium CC	conditioned medium MCS	conditioned medium MCF	conditioned medium mat. control
**glass control**	1	0.97 ± 0.04	1.01 ± 0.25	0.99 ± 0.01	1.00 ± 0.00
	3	0.96 ± 0.02	6.41 ± 4.07 ****	1.03 ± 0.01	1.00 ± 0.00
	7	0.99 ± 0.02	1.19 ± 0.10	1.03 ± 0.02	1.00 ± 0.00
**Mg**	1	1.00 ± 0.01	1.01 ± 0.02	1.00 ± 0.01	1.02 ± 0.03
	3	0.97 ± 0.03	0.98 ± 0.03	0.99 ± 0.02	0.99 ± 0.04
	7	0.98 ± 0.02	0.89 ± 0.09	0.99 ± 0.04	1.02 ± 0.04
**Mg–6Ag**	1	1.00 ± 0.01	1.01 ± 0.03	1.00 ± 0.01	1.01 ± 0.04
	3	0.96 ± 0.03	0.98 ± 0.04	1.01 ± 0.02	0.99 ± 0.07
	7	0.99 ± 0.01	0.89 ± 0.05	1.02 ± 0.02	1.02 ± 0.03

#### 3.2.2 Mg exposition affected endothelial cell proliferation, migration and tube formation

The next step of the angiogenesis is initiated by an increased endothelial cell proliferation and migration. Therefore, the HUVEC proliferation and migration was monitored with respect to cell culture medium released from cocultures under the impact of degradable Mg-based material.

Fig. 8a summarizes the proliferation of the HUVEC indicated by normalized fluorescence intensities of cell nuclei staining directly after changing to the conditioned media (day 0) and after three and seven days. A steady HUVEC proliferation was observed on the glass control for all conditions, with a minor proliferation increase with just cell culture medium (mat. control). Incubation of the HUVEC with conditioned media that were obtained from cells grown on pure Mg (CC, MCS, MCF) led to significant increase in endothelial cell proliferation from day 0 to day 3 and a subsequent decrease to day 7. However, only slight cell number changes were observed with conditioned media produced on Mg–6Ag.

**Fig. 8a:**
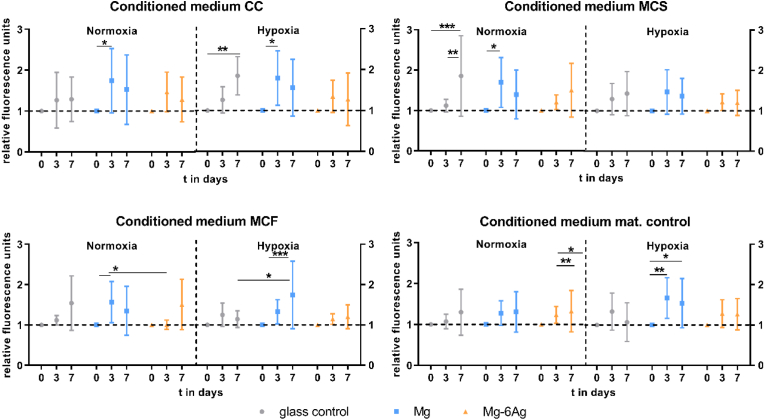
Endothelial cell proliferation with different conditioned media. HUVEC were incubated with conditioned media from the coculture (CC) or Saos-eGFP (MCS) and RF Fibroblasts (MCF) in monocultures on Mg-based materials or material without cells. Fluorescence intensities (normalized to day 0) are shown as the mean ± SD from two experiments with six triplicates (n = 12). Statistics: two-way ANOVA (materials, time points; compared to day 0) with Tukey's multiple comparison test. * = p < 0.05; ** = p < 0.01; *** = p < 0.001.

**Fig. 8b:**
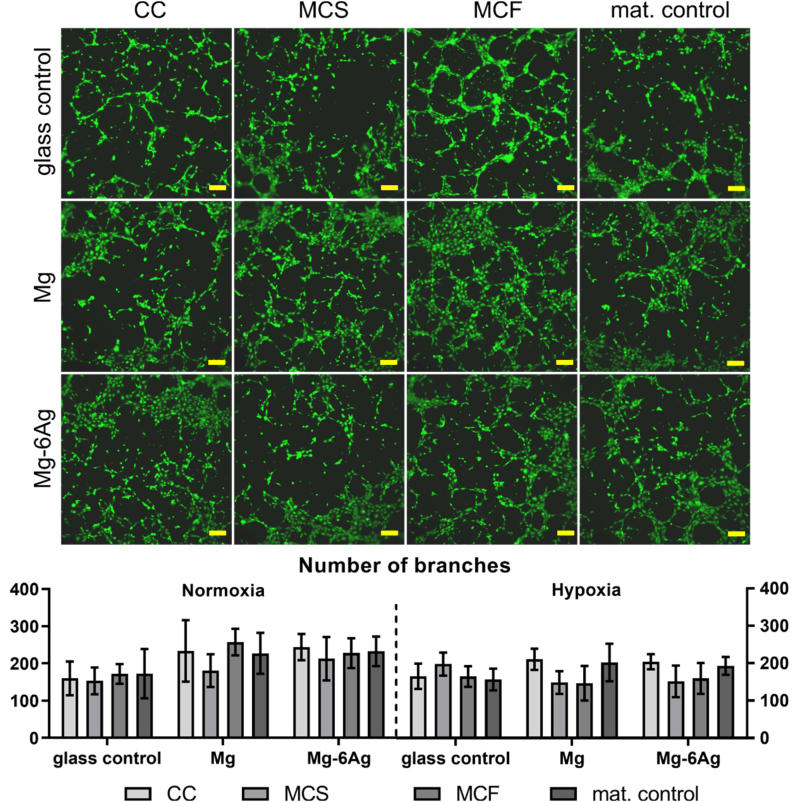
Endothelial cell tube formation. Representative pictures of HUVEC capillary structures under normoxia stained with calcein-AM. Scale bar is 100 μm. Images were analyzed with the “Angiogenesis analyzer” from ImageJ and branch numbers are shown as the mean ± SD from two experiments with three samples (n = 6). Statistics: two-way ANOVA (materials, time points) with Tukey's multiple comparison test.

**Fig. 9:**
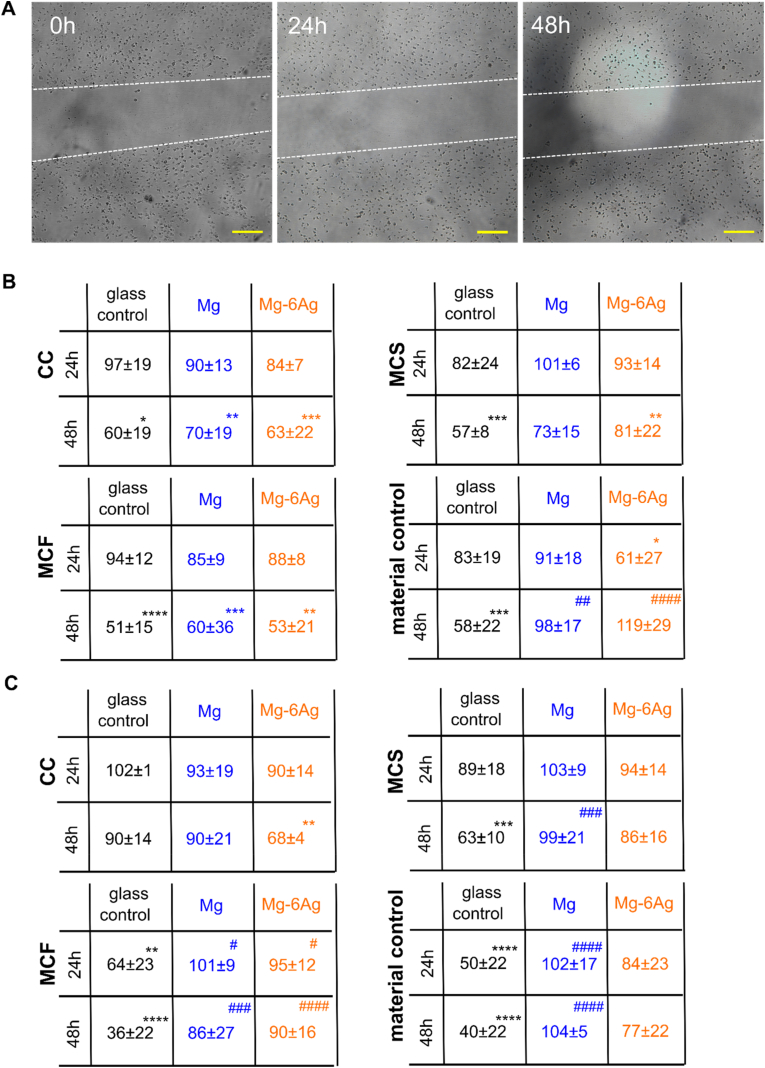
Endothelial cell migration with different conditioned media. HUVEC were incubated with conditioned media from the coculture (CC) or Saos-eGFP (MCS) and RF Fibroblasts (MCF) in monocultures on Mg-based materials or material without cells. (A) Representative microscopic images of the scratch area in a HUVEC layer within 48 h. The white dotted lines symbolize the cell fronts. Scale bar is 100 μm. (B, C) Quantification of the scratch area in relation to the initial cell-free area under normoxia (B) and hypoxia (C). Relative cell-free areas are shown as the mean ± SD from two experiments with two samples and three chosen positions (n = 12). Statistics: two-way ANOVA (Mg, Mg–6Ag compared to glass control = #; 24 h, 48 h compared to 100% at 0 h = *) with Tukey's multiple comparison test. One symbol = p < 0.05; two symbols = p < 0.01; three symbols p < 0.001; four symbols = p < 0.0001.

Fig. 9 shows the migration of HUVEC (Fig. 9A) after the incubation with the different conditioned media for up to 48 h under normoxia (Fig. 9B) and hypoxia (Fig. 9C).

The scratch area was significantly reduced within 48 h, when HUVEC were exposed to the conditioned medium from the glass control (Fig. 9B). Comparable results were obtained for conditioned media from Mg and Mg–6Ag with no significant difference to the glass control. However, the use of conditioned media that was produced only with Mg and Mg–6Ag without cells indicated an averagely constant relative scratch area in the HUVEC layer. Therefore, those two conditions (Mg, Mg–6Ag, no cells) led to a significant reduction of HUVEC migration compared to the glass control.

The reduction of O_2_ level to hypoxic conditions slowed down the endothelial cell migration after incubation with conditioned media related to the coculture (Fig. 9C). Nevertheless, the scratch area significantly reduced within 48 h with the conditioned media of the glass control seeded with the monocultures or without cells comparable to normoxic conditions. However, the migration appeared to be significantly reduced compared to the glass control for the Mg-based materials.

The HUVEC tube formation is depicted and quantified in Fig. 8b.

Incubating HUVEC with the conditioned media that relate to the Mg-based materials tended to increased branch numbers under normoxic conditions irrespective of the cell type combination (coculture, monoculture). Contrary to that, conditioned media of the monocultures of tumor and healthy cells on pure Mg tended to reduce the branch number in the capillary network under hypoxia.

## 4 Discussion

In this study, we investigated aspects of the potential suitability of Mg-based materials as a novel local anticancer therapy approach to prevent the occurrence and progression of cancer metastases. To analyze the effects on cell migration and invasion we used an osteosarcoma-fibroblasts coculture model that has succeeded as a test system for the anticancer activity of pure Mg and Mg–6Ag materials [12].

We measured a Mg concentration during Mg material degradation between 2.5 (day 1) and 4.0 (day 7), which was in an expected range [[31], [32], [33], [34], [35]]. The increased Mg concentration from degrading Mg-based materials can have an important impact on the cell physiology and metabolism. Mg-ions act as a stabilizer and protector for the DNA structure and as cofactors for DNA replication and repair enzyme systems [36]. This underlines the character of avoiding the formation of cancer cells (nox-induced mutations, deficient repair system). However, Mg-ions are also involved in energy metabolism by their cofactor function in glycolytic enzymes or as stabilizers of adenosine triphosphate (ATP) [37]. This indicates an ambiguous function of Mg-ions in carcinogenesis.

Furthermore, we measured the Ag concentration in the supernatant during degradation of Mg–6Ag (around 0.2 μM). Noorbazargan et al. showed a reduced cancer cell migration and invasion with Ag materials in their work [38]. Ag can also further influence cells as follows: Ag can induce cancer cell death via p53 and subsequent DNA damage and suppression of NF- κB [39]. It can also reduce LDH activity, major enzyme in lactate fermentation used to generate energy in cancer cells, and subsequently induces caspase-dependent apoptosis. Furthermore, Ag increases super oxide dismutase activity, while catalase and glutathione peroxidase are not affected. This can lead to a redox imbalance and elevated H_2_O_2_ levels. Healthy cells are less sensitive to H_2_O_2_ due to an intact antioxidant defense [40]. Additionally, Ag may reduce the expression of proinflammatory cytokines (TNF-α, IL-12, VEGF) in fibroblasts [41].

The supernatants were also used as conditioned media to incubate the HUVEC with this. Ag-ions majorly influence the cell viability but can also diminish the membrane integrity in endothelial cells [42]. Guo and colleagues also reported a reduced endothelial layer integrity accompanied with an increased level of reactive oxygen species in HUVEC cells [43]. The increased endothelial layer integrity might promote the cancer induced angiogenesis as an initial step. However, the authors showed that Ag nanoparticles were predominately internalized rather than Ag-ions. This hints to the awareness that the added Ag in the Mg-based materials probably does not influence the cancer-induced angiogenesis. However, Ag is known to act antibacterial, which can reduce implant associated infections and in consequence potentially avoids surgical revision.

To investigate the effect of degradable Mg-based materials on cancer metastases, the cell migration and cell invasion of the coculture was analyzed. This revealed a significant reduction of migration and invasion for the coculture in presence of Mg and Mg–6Ag in comparison to a non-degradable glass control. In contrast to this, the migration inhibition was less pronounced when the monocultures were investigated. This indicated an essential communication between cancer cells (Saos-eGFP) and host fibroblasts (RF Fibroblasts) that ultimately led to an inhibited cell migration only with degradable Mg-based materials, whereas the fibroblasts play a crucial role in migration inhibition [44,45]. Similar results were obtained for the cell invasion, where Mg and Mg–6Ag significantly reduced the cell invasion of the coculture. The results for the cell migration and invasion are in accordance with the results from Wu et al. [46]. In their study, the authors observed a reduced cell migration and invasion for the osteosarcoma cell line U2OS, when they incubated the cells with extracts obtained from Mg-based materials. The authors explained this with increased levels of phosphorylated, thus activated ERK1/2, JNK and p38 from MAPK signaling. However, other publications found decreased cell migration and invasion by inhibiting the MAPK signaling [47], or in accordance with a decreased p-ERK1/2 and p-p38 expression level [48,49]. This suggests different signaling pathways that are involved in the reduced cell migration and invasion with Mg-based materials, such as an altered MMP activity.

Those MMPs, especially MMP-2 and MMP-9, are driving forces for cancer cell invasion and metastases formation [50,51]. During the metastatic cascade, cancer cells can exploit fibroblasts to secret MMPs to facilitate cancer cell invasion by ECM decomposition [52,53]. Though as shown previously, the RF fibroblasts in the presented osteosarcoma-fibroblasts coculture retained their normal phenotype and did not undergo changes to cancer-associated fibroblasts [54]. In our work, the degradation of the here used Mg-based materials led to an increased secretion of MMP-2 and MMP-9 and suggested an increased invasion and metastatic capability of osteosarcoma cells under the impact of those materials. Though, this can be explained by the results of our previous work [12], where we showed an increased fibroblast cell number ratio in the coculture on the investigated Mg-based materials. With the fact that fibroblasts are a main source of MMP secretion, this will lead to increased MMP concentrations in the supernatant. This result is supported by several publications, where the authors showed increased MMP-2 levels, when they incubated cancer cells with fibroblasts in a coculture compared to these cell types seeded alone [[55], [56], [57]]. However, also the TIMP-1 secretion was increased under these conditions. TIMP-1 is a natural antagonist of MMPs e.g. MMP-2 and MMP-9 [58,59], as it binds MMPs in a 1:1 stoichiometric ratio and impedes the binding of substrates to the catalytic domain of the MMPs [60].

Not only the pure difference in cell ratios on degradable Mg-based materials and the non-degradable control, differences in MMP and TIMP-1 secretion can be explained by the Mg material degradation. In contrast do our results, it was already shown that the expression of MMP-2 and MMP-9 were increased under Mg deficiency [61,62]. Additionally, several authors indicated increased MMP-9 secretion and activity due to the acidic TME, with decreasing activity at a neutralized extracellular pH (pH_e_) [[63], [64], [65]]. Conversely, the TIMP-1 activity is low under the influence of an acidic pH_e_ but increases with an increasing pH_e_ [66]. This can explain the increased TIMP-1 secretion and activity during Mg material degradation, while the MMP activity was relatively low at alkaline pH_e_ In general, the acidity of the TME appears to be an important factor that promotes tumor invasion and metastases [[67], [68], [69]] and the pH neutralization with an oral administered alkaline agent was shown to inhibit cancer metastases [70,71]. Aside from the alkaline pH_e_, molecular hydrogen was already shown to exert antitumor activity [[72], [73], [74]]. Liu et al. (2019) and Shang et al. (2018) showed a decreased cell migration and invasion for glioblastoma and ovarian cancer cells due to the incubation with molecular hydrogen, whose evolution is also relevant during Mg material degradation [75,76]. Therefore, despite the increased MMP concentration (maximum of 2 mM, see Fig. 5), a further increased TIMP-1 secretion (maximum of 10 mM, see Fig. 5) resulting from Mg material degradation will ultimately lead to the inhibition of MMP activity and therefore opens the opportunity for a reduced invasion and metastases.

Angiogenesis is a critical process to allow a sufficient nutrition and oxygenation of the tumor to promote tumor growth, tumor progression and to support invasion and metastases. To investigate the effects of Mg-based material degradation on cancer-induced angiogenesis, we applied conditioned media of the tumor and healthy cells grown on Mg-based materials under normoxia and hypoxia. The supernatants of the cell-seeded immersed materials were harvested and later incubated with the HUVEC to simulate the interaction and molecular communication in a tumor microenvironment and to investigate the cancer-induced angiogenesis. This experimental set-up ensured the relevance of the obtained results by targeting the effects of both cytokines as well as Mg degradation products. One the other side, this set-up avoided the difficulties of a direct triple culture (Saos-eGFP, RF Fibroblasts, HUVEC) with different optimal culture conditions and visualization issues on opaque material, thus keeping the cell system effective.

The increase of endothelial cell layer permeability is the initial step of angiogenesis [77]. Our results showed an increased HUVEC layer permeability under incubation with the conditioned medium of the glass control compared to the conditioned media of cell-related Mg-based materials (CC, MCS, MCF). Similar results were presented by Zhu and colleagues [78]. The authors measured a Mg-dependent transmonolayer electrical resistance of the endothelial cell layer and showed that a Mg treatment reduced the endothelial cell layer permeability in comparison with Mg deficiency. They resumed that S1P1‐Rac1 pathways are involved in the enhanced barrier integrity as well as barrier stabilizers such as cyclic adenosine monophosphate (cAMP), fibroblast growth factor (FGF) and endothelial nitric oxide synthase (eNOS), leading to interendothelial tight junction assembly [[78], [79], [80]]. On the other side, VEGF is a mediator of endothelial layer permeability. Our results showed that the monoculture of Saos-eGFP significantly increased the VEGF secretion, while Mg-based materials did not significantly influence the VEGF protein level. This is in accordance with the results from Zhu and colleagues, who found an increased VEGF expression on mRNA level with Mg treatment but no changes on protein level [78]. The permeability is also affected by other Mg-degradation dependent surface-near effects. Zougbédé et al. (2011) found that the permeability of microvascular endothelial cells was increased under an acidic pH, whereas it decreased with alkalinity [81]. Lastly, Xie et al. (2015) showed that a treatment with hydrogen-rich water increased the transendothelial electrical resistance and endothelial cadherin expression, and therefore, reduced the endothelial layer permeability [82]. Thus, Mg material degradation can reduce endothelial cell layer permeability by an increased Mg concentration, alkaline pH, and hydrogen effect to reduce cancer-induced angiogenesis already at an early step.

VEGF is further involved in angiogenesis as a main mediator of endothelial cell proliferation and migration. Our results indicated an increase in HUVEC proliferation (material unspecific) under the influence of cell-related conditioned media of the Mg-based materials and the glass control. Though, conditioned media of the Mg materials without cells (mat. control) did not lead to changes in the HUVEC proliferation in comparison with the glass control. This suggests that the Mg material degradation is not responsible for endothelial cell proliferation but the seeded cancer cells and fibroblasts that can secret proliferation promoting cytokines [83]. However, the role of Mg degradation and extracellular Mg concentrations in endothelial cell proliferation are further ambiguously discussed in the literature with publications indicating a proliferation stimulation [78,[84], [85], [86]] rather than an inhibiting function [87,88]. Moreover, our results indicated a decreased endothelial cell migration under the influence of conditioned media obtained from the used Mg-based materials without cells (material control). The cancer cells and fibroblasts in coculture or monocultures appeared to reduce this migration inhibition again. Contrary to our results, previous studies showed an enhanced endothelial cell migration with Mg supplementation [78,84,87,88]. However, also the other Mg material degradation dependent surface-near effects can affect endothelial migration in a direct *in vitro* cell model or *in vivo*, respectively. A decreased pH_e_, as observed in the TME, was previously associated with an inhibited endothelial cell proliferation and migration [89,90]. Therefore, it was assumed that an increased pH during Mg material degradation will also decrease the HUVEC migration, contrary to our results.

Lastly, we found that the conditioned media that was produced with cocultured cells on Mg-based materials increased the tube formation of HUVEC under both normoxia and hypoxia, which was in accordance with findings from Gao et al. (2020) [91]. Though, the ability to form tube-like structures seemed to be not affected under hypoxic conditions. Since cancer cells predominantly induce angiogenesis under hypoxic conditions, we assume that the increased Mg concentration in the conditioned media from the degradation of the here used materials do not influence the assembly of HUVEC into capillary structures. However, a direct experimental set-up is required to determine the effect of the complex Mg degradation or an approach with the individual surface-near effects to detect their individual impacts.

In summary we can say that the increased Mg concentration from the degradation of the used Mg-based materials can potentially diminish the cancer-induced angiogenesis at an early stage. While the endothelial cell layer integrity seems to be enhanced by the products of Mg-based material degradation, the tube formation was not affected. Future studies should focus on the influence of the other surface-near effects on cancer cell migration and invasion, as well as cancer-induced angiogenesis.

## 5 Conclusion

Our results suggest Mg-based materials as promising materials with an anticancer activity and therefore suitable tools for an osteosarcoma therapy. The materials inhibited cell migration and invasion and can potentially reduce the cancer-induced angiogenesis at an early step. This diminishes the probability of metastases and tumor progression during a potential therapy with Mg-based materials. However, the degradation of the Mg-based materials is the central parameter that determines the environmental changes (pH, osmolality, Mg concentration) and consequently our results depict the influence of slow degrading Mg-based materials on the osteosarcoma progression. Hence, future studies should verify these results using materials with different degradation rates. Medium solutions, altering one parameter of the surface-near effects during Mg material degradation may allow to identify the influencing effect (pH, osmolality, Mg concentration, H_2_) that is responsible for reduced tumor progression and suggests possible signaling pathways involved.

## Funding

This work was funded by the 10.13039/501100006769Helmholtz – Russian Science Foundation Joint Research Groups HRSF-0025.

## Ethical statement

All experiments were performed in accordance with German ethical regulations.

## CRediT authorship contribution statement

**Philipp Globig:** Conceptualization, Formal analysis, InvestigationInvestigation, Methodology, Validation, Visualization, Writing – original draft, Writing – review & editing. **Roshani Madurawala:** InvestigationInvestiagtion, Validation, Visualization. **Regine Willumeit-Römer:** Conceptualization, Funding acquisition, Project administration, Supervision, Writing – review & editing. **Fernanda Martini:** Resources. **Elisa Mazzoni:** Resources. **Bérengère J.C. Luthringer-Feyerabend:** Conceptualization, Funding acquisition, Methodology, Project administration, Supervision, Writing – review & editing.

## Declaration of competing interest

The authors declare no conflict of interest. The funders had no role in the design of the study; in the collection, analyses, or interpretation of data; in the writing of the manuscript, or in the decision to publish the results.

## Figures and Tables

**Table A4:** VEGF release under normoxia. HUVEC were incubated with conditioned media from the coculture (CC) or Saos-eGFP (MCS) and RF Fibroblasts (MCF) in monocultures on Mg-based materials or material without cells. Fold changes of VEGF concentration (relative to cell culture medium) are shown as the mean ± SD from two experiments with three samples (n = 6). Statistics: one-way ANOVA (sample vs medium control) with Dunn's multiple comparison test. **** = p < 0.0001.

	Day	conditioned medium CC	conditioned medium MCS	conditioned medium MCF	conditioned medium mat. control
**glass control**	1	1.00 ± 0.01	1.01 ± 0.02	1.00 ± 0.01	1.00 ± 0.00
	3	0.93 ± 0.03	1.27 ± 0.20	1.04 ± 0.09	1.00 ± 0.00
	7	0.93 ± 0.06	0.97 ± 0.02	0.96 ± 0.04	1.00 ± 0.00
**Mg**	1	1.00 ± 0.01	1.00 ± 0.01	1.00 ± 0.01	1.07 ± 0.16
	3	0.91 ± 0.02	1.17 ± 0.23	0.97 ± 0.02	1.01 ± 0.07
	7	0.92 ± 0.01	0.88 ± 0.06	0.93 ± 0.02	0.96 ± 0.04
**Mg–6Ag**	1	0.99 ± 0.00	1.00 ± 0.02	0.99 ± 0.01	1.01 ± 0.02
	3	0.92 ± 0.01	1.26 ± 0.20	0.99 ± 0.05	1.03 ± 0.07
	7	0.92 ± 0.03	0.94 ± 0.04	0.95 ± 0.05	0.96 ± 0.06
